# Refractory hemobilia following liver transplantation

**DOI:** 10.1055/a-2964-6832

**Published:** 2026-09-30

**Authors:** Songming Ding, Xiangyan Liu, Yang Liu, Yifei Wu, Shusen Zheng, Qiyong Li, Wu Zhang

**Affiliations:** 1 Division of Hepatobiliary and Pancreatic Surgery 636046 Shulan Hangzhou Hospital Affiliated to Zhejiang Shuren University, Shulan International Medical CollegeHangzhouZhejiangChina; 2 Endoscopy Center 636046 Shulan Hangzhou Hospital Affiliated to Zhejiang Shuren University, Shulan International Medical CollegeHangzhouChina

Hemobilia is a rare but potentially fatal cause of upper gastrointestinal bleeding. 1​ Timely diagnosis and precise localization of the bleeding source remain significant clinical challenges. 2​ ​3​ We report a case of refractory hemobilia following liver transplantation (LT), which was ultimately controlled via common hepatic artery embolization after multiple failed interventions.

A 74-year-old woman underwent split LT 3 months prior for hepatocellular carcinoma complicated by decompensated hepatitis B cirrhosis. Early postoperative complications included portal vein thrombosis with subsequent occlusion (managed with stenting) and a bile leak (treated with endoscopic retrograde cholangiopancreatography [ERCP]; Fig. 1 ).

**Fig. 1:**
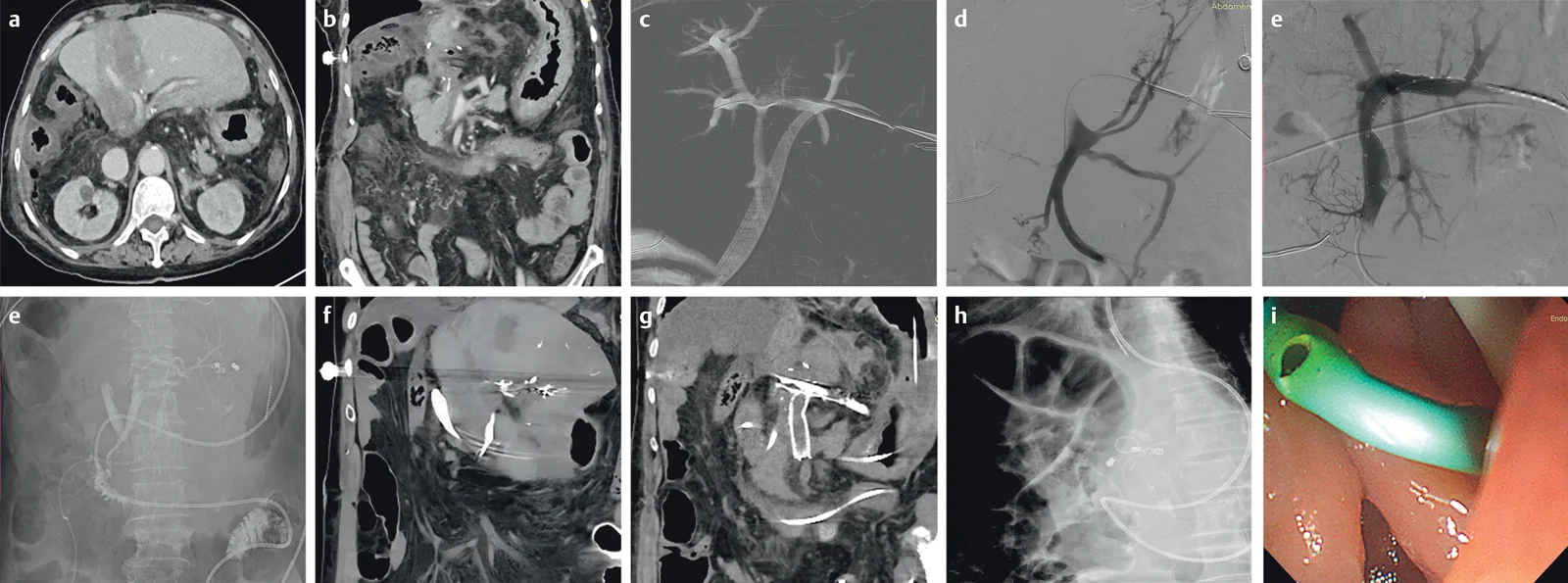
Management of post-liver transplantation complications. ( **a** and **b** ) Contrast-enhanced abdominal CT revealed portal vein thrombosis. ( **c** ) Ultrasound-guided portal vein puncture and angiography were performed. ( **d** ) Superior mesenteric vein angiography was performed. ( **e** ) A metallic portal vein stent was placed. ( **f** ) T-tube cholangiography indicated a post-transplant bile leak. ( **g** and **h** ) Non-contrast CT demonstrated contrast extravasation extending to the perihepatic space and into the abdominal drainage tube. ( **i** and **j** ) A 5 F×9 cm plastic biliary stent was placed via ERCP. CT, computed tomography; ERCP, endoscopic retrograde cholangiopancreatography.

Nearly 2 months after LT, the patient presented with abdominal distension, melena, and a significant drop in hemoglobin (from 139 to 104 g/L). Gastroscopy revealed bloody fluid oozing from the duodenal papilla. Angiography identified a pseudoaneurysm of the pancreaticoduodenal artery, which was managed with coil embolization ( Video 1 ). Bleeding recurred 3 days later. Although subsequent angiography was negative, emergency ERCP confirmed hemobilia, and a fully-covered self-expandable metal stent was placed ( Fig. 2 ).

**Video 1** Combined vascular interventional and endoscopic management of this refractory hemobilia.


**Fig. 2:**
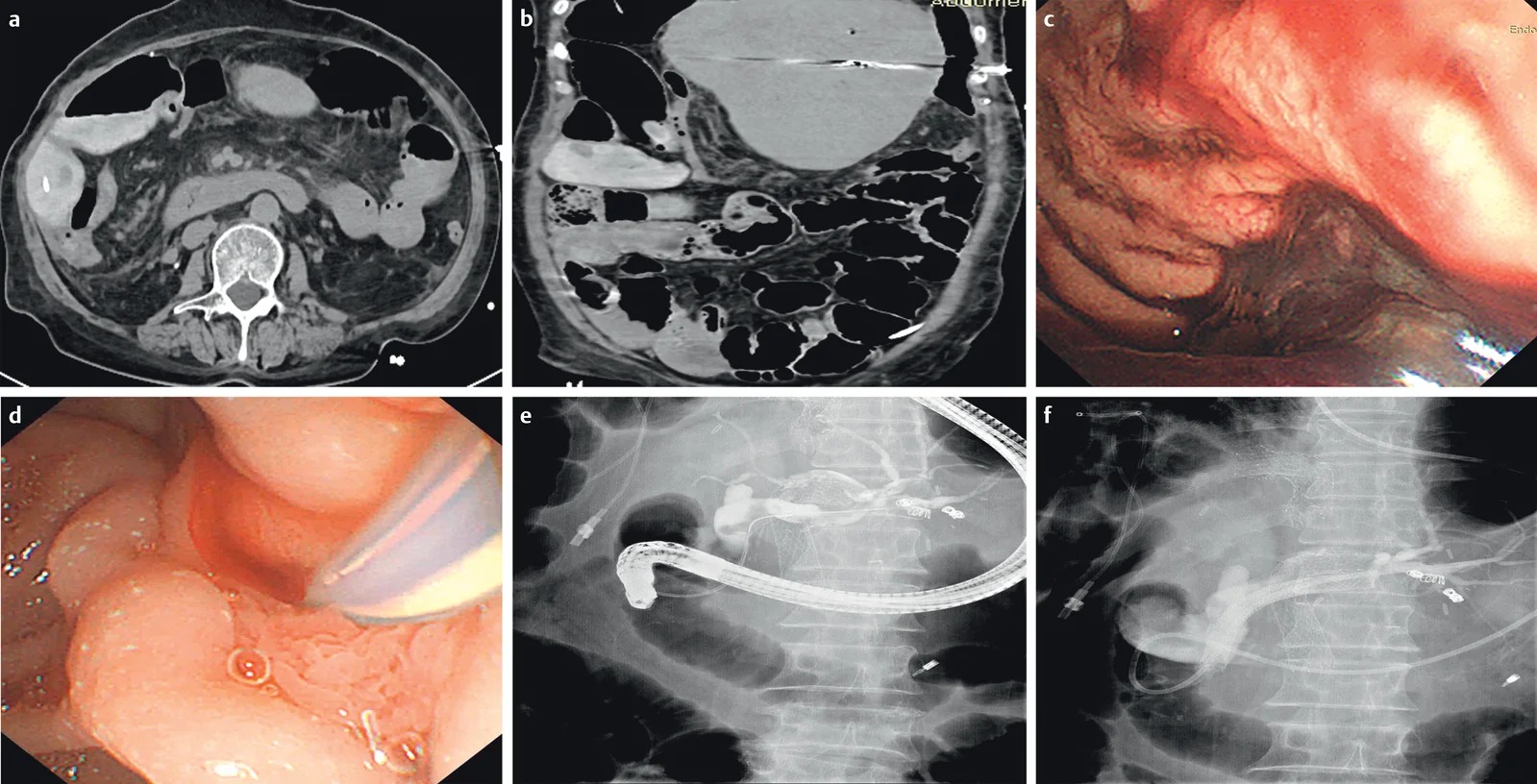
Diagnosis and endoscopic management of hemobilia. ( **a** and **b** ) Non-contrast abdominal CT revealed gas and fluid accumulation in partial bowel loops, along with high-density opacities within the intestinal lumen, suggesting gastrointestinal bleeding. ( **c** ) Duodenoscopy identified dark red blood clots in the gastric cavity. ( **d** ) After successful biliary cannulation, bloody fluid was observed oozing from the bile duct. ( **e** ) Blood clots were observed in the bile duct. ( **f** ) A fully-covered metal stent was placed, and an ENBD tube was inserted. ENBD, endoscopic nasobiliary drainage.

Twenty days later, recurrent hemorrhage occurred, with the hemoglobin level decreasing from 62 to 51 g/L. Although liver computed tomography angiography revealed no obvious vascular malformations, selective embolization of the hepatic artery and portal vein branches was performed empirically, given the patient's history of ultrasound-guided portal vein puncture ( Video 1 ). Five days later, melena recurred; ERCP revealed stent migration, prompting its removal ( Fig. 3 ). Cholangioscopy showed a congested bulge in the recipient bile duct ( Video 1 ). A fourth angiography identified and embolized a pseudoaneurysm of the common hepatic artery. Following sudden hematemesis 7 days later, a fifth angiography achieved the complete embolization of the common hepatic artery ( Video 1 ). Hemoglobin levels recovered to 97 g/L by discharge. At the 2-week follow-up, the hemoglobin level remained stable at 96 g/L, with total bilirubin within normal limits.

**Fig. 3:**
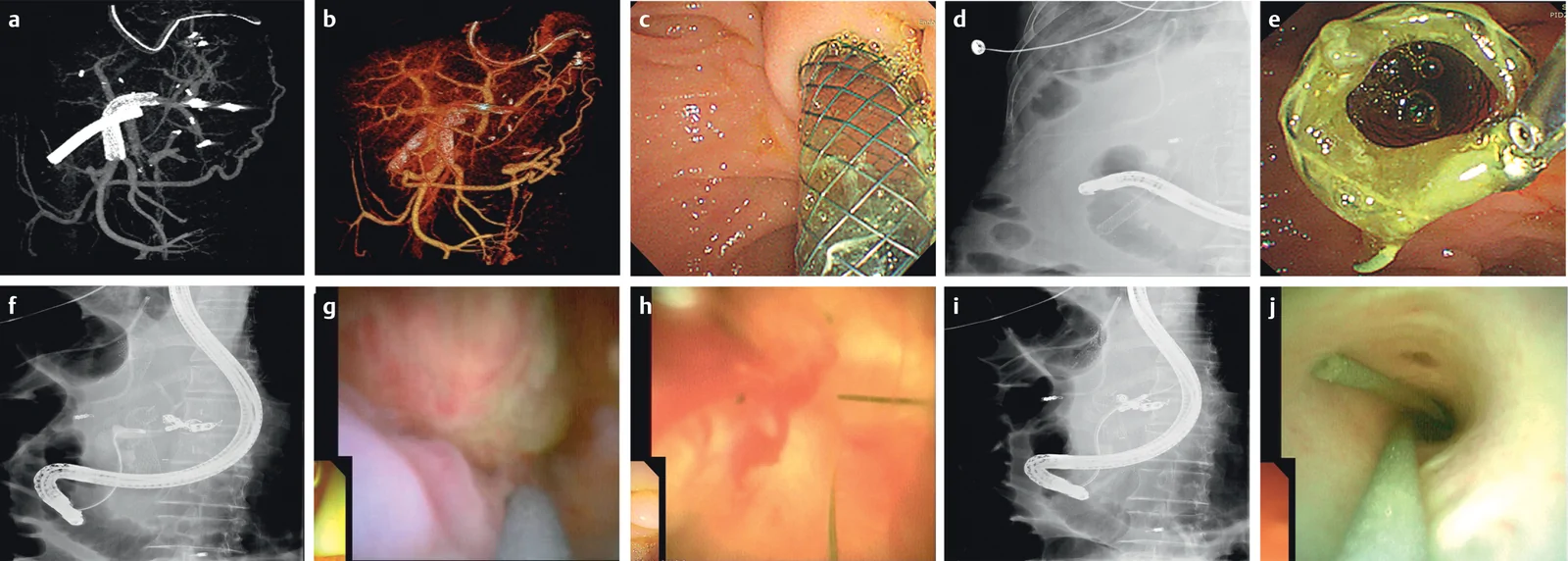
Liver CTA to evaluate problematic vasculature and peroral cholangioscopy to explore the biliary tract. ( **a** and **b** ) Liver CTA revealed no obvious abnormal vessels. ( **c** and **d** ) The metallic stent was found to have migrated below the anastomosis. ( **e** ) The stent was successfully removed using biopsy forceps. ( **f** and **g** ) Peroral cholangioscopy demonstrated a congested bulge in the recipient bile duct. ( **h** ) Sutures at the anastomosis with minor blood clots attached. ( **i** and **j** ) Peroral cholangioscopy of the intrahepatic bile ducts showed no active bleeding sites. CTA, computed tomography angiography.

This case of refractory hemobilia post-LT may be associated with prior vascular interventions for portal vein occlusion, while bile leaks may also serve as predisposing factors for hepatic artery pseudoaneurysm formation. Endoscopic failure was partially attributed to the spontaneous migration of the metal stent. 4​ Although main hepatic artery embolization successfully achieved hemostasis, the patient requires rigorous long-term monitoring for ischemic cholangiopathy. 5​

UCTN_Code_CPL_1AK_2AI

## Declaration of GenAI Use

The authors confirm that no generative artificial intelligence was used in the preparation of this manuscript.
